# In vivo neuroprotective capacity of a *Dunaliella salina* extract - comprehensive transcriptomics and metabolomics study

**DOI:** 10.1038/s41538-023-00246-7

**Published:** 2024-01-10

**Authors:** Alberto Valdés, José David Sánchez-Martínez, Rocío Gallego, Elena Ibáñez, Miguel Herrero, Alejandro Cifuentes

**Affiliations:** Laboratory of Foodomics, Institute of Food Science Research (CIAL, CSIC-UAM), Calle Nicolás Cabrera 9, 28049 Madrid, Spain

**Keywords:** Mass spectrometry, RNA, Metabolomics

## Abstract

In this study, an exhaustive chemical characterization of a *Dunaliella salina* (DS) microalga extract obtained using supercritical fluids has been performed, and its neuroprotective capacity has been evaluated in vivo using an Alzheimer’s disease (AD) transgenic model of *Caenorhabditis elegans* (strain CL4176). More than 350 compounds were annotated in the studied DS extract, with triacylglycerols, free fatty acids (FAs), carotenoids, apocarotenoids and glycerol being the most abundant. DS extract significantly protects *C. elegans* in a dose-dependent manner against Aβ-peptide paralysis toxicity, after 32 h, 53% of treated worms at 50 µg/mL were not paralyzed. This concentration was selected to further evaluate the transcriptomics and metabolomics changes after 26 h by using advanced analytical methodologies. The RNA-Seq data showed an alteration of 150 genes, mainly related to the stress and detoxification responses, and the retinol and lipid metabolism. The comprehensive metabolomics and lipidomics analyses allowed the identification of 793 intracellular metabolites, of which 69 were significantly altered compared to non-treated control animals. Among them, different unsaturated FAs, lysophosphatidylethanolamines, nucleosides, dipeptides and modified amino acids that have been previously reported as beneficial during AD progression, were assigned. These compounds could explain the neuroprotective capacity observed, thus, providing with new evidences of the protection mechanisms of this promising extract.

## Introduction

Dementia is currently the seventh leading cause of death among all diseases and one of the major causes of disability and dependency among older people globally^1^. Alzheimer’s disease (AD) is the leading and most common form of dementia, with 60-70% cases reported worldwide^1^. AD is characterized by a progressive loss of neurons from different regions of the brain, causing cognitive decline, brain atrophy, loss of cholinergic neuronal activity and mental deterioration^2^. Histopathological features of AD consist of aggregates of hyperphosphorylated tau protein and amyloid beta (Aβ) peptide, which form intracellular neurofibrillary tangles (NFTs) and extracellular senile plaques (SPs), respectively; but also the decline of the cholinergic system, large oxidative stress and neuroinflammation, among other hallmarks^3,4^. Moreover, recent research studies have suggested that altered metabolism of lipids, vitamins, L-arginine or nitric oxide (NO) may be involved in the pathogenesis and development of AD^5–8^.

Although the knowledge about the genetic factors contributing to AD has greatly evolved throughout the years to understand the pathological process of AD^9^, the only approved drugs for AD treatment are cholinesterase inhibitors and an antagonist of the N-methyl-D-aspartate receptor, which can inhibit the symptoms but not reverse AD progression^10^. In addition, these drugs have several side effects and other alternatives are being searched. These new approaches are focused on the modification of lifestyle factors, such as diet^11^, or the investigation of nutraceuticals that could prevent or retard AD occurrence^12^. These dietary and natural components include carotenoids, omega-3 fatty acids, fat-soluble vitamins, terpenoids or phenolic compounds, among others, which may interfere with different molecular mechanisms related to AD development, including protection against Aβ plaque formation and aggregation^13^. Among these dietary sources, microalgae have gained an increased interest in the last decade, such as the case of *Dunaliella salina*. This microalga is rich in carotenoids, which have been demonstrated to play an important role protecting the cellular components against reactive oxygen species (ROS)^14^, but also to act as modulators of inflammation-related mechanisms^15,16^. Moreover, carotenoids can also enhance the endogenous antioxidant systems^17^.

In a previous study, we have demonstrated the antioxidant, anti-inflammatory and anti-cholinergic capacities of a carotenoid-enriched extract from *D. salina* obtained by supercritical fluid extraction (SFE), and also its neuroprotective effect in the human neuron-like SH-SY5Y cell model^18^. The comprehensive lipidomics/metabolomics study carried out allowed the identification of significantly increased phosphatidylcholines (PCs), triacylglycerols (TGs) and fatty acids (FAs) after the treatment with the extract, while several phosphatidylglycerols (PGs) were found to be decreased, which could contribute to the observed neuroprotection. However, to corroborate the in vitro findings and to expand our knowledge on the metabolic pathways involved in the progression and prevention of AD, in vivo models are needed. Among these models, *Caenorhabditis elegans* is an invertebrate organism with unique advantages, such as a short lifespan, well-characterized nervous system and genome sequence, and it has been highly investigated in developmental biology, neurobiology and aging^19^. It has also been widely employed in neuroscience-related studies of AD^20^, including therapy test with drugs and natural extracts^21–23^. For instance, the antioxidant potential of individual carotenoids (some of which are present in *D. salina*), such as astaxanthin^24^, lutein^25^, β-carotene, lycopene and β-cryptoxanthin^26^, or food matrices enriched in carotenoids, such as orange juices^27–29^, have been demonstrated on *C. elegans*.

There has also been an increased interest in analyzing the metabolite and lipidic composition of this nematode (recently reviewed)^30^, and new analytical approaches have been developed to expand the metabolome/lipidome coverage of this organism^31,32^. These methodologies have been used to investigate the relationship between ageing, longevity and metabolism (associated to specific genes, such as *daf-2*, *eat-2* or *slcf-1*). In other cases, signaling molecules or lipids have been targeted and extensively investigated; and to a lesser extent, the food source and nutrition effects on *C. elegans* metabolome^30^. In other works, metabolomics, transcriptomics and computational modeling have been integrated to evaluate the metabolic stress in a *C. elegans* model expressing pan-neuronal human Aβ peptide^33^, or to investigate the metabolic changes that occur during aging^34^. However, a comprehensive metabolomics and lipidomics study combined with advanced RNA-Seq analysis of a *C. elegans* model fed with a carotenoids-enriched extract has never been accomplished.

Based on the previous information, the main goal of the present study was to perform an exhaustive chemical characterization of a *D. salina* microalga extract (DS) obtained by SFE, to evaluate its in vivo neuroprotective capacity using an AD transgenic model of *C. elegans* (CL4176), and to investigate the transcriptomics and metabolomics changes produced after the treatment by using advanced methodologies (RNA-Seq and GC/LC-MS technologies).

## Results

### Phytochemical characterization of *Dunaliella salina* extract

**Fig. 1:**
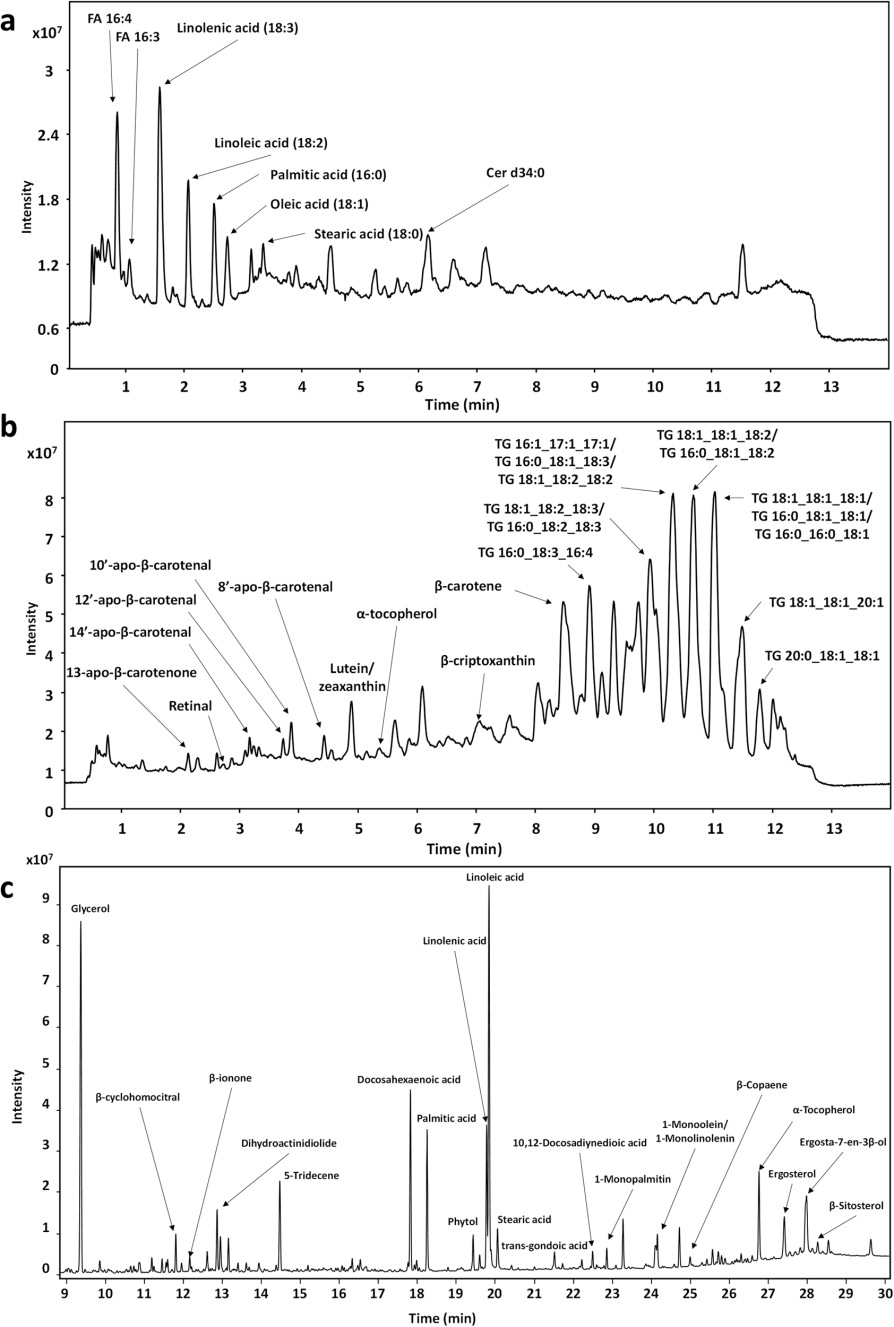
Total ion current (TIC) chromatograms and the most abundant annotated compounds in *Dunaliella salina* (DS) extract. **a** CSH-Q-TOF MS/MS ESI (-) analysis. **b** CSH-Q-TOF MS/MS ESI (+) analysis. **c** GC-Q-TOF MS analysis.

To get a comprehensive view on the chemical composition of the DS extract, two complementary untargeted metabolomics approaches were performed: CSH-Q-TOF MS/MS and GC-Q-TOF MS. The analysis performed by CSH-Q/TOF MS/MS resulted in the annotation of 48 compounds in ESI (-) and 192 in ESI (+), giving a total of 240 annotations. Among them, the most remarkable lipids in ESI (-) were FAs (44 species), but two acylhexosyl campesterols (ASG 28:1;O;Hex;FA 16:0 and ASG 28:1;O;Hex;FA 18:1), one PE (34:1) and one ceramide (d34:0) were also identified (Supplementary Table 1). The most intense peaks corresponded to FA 18:3 (linolenic acid), FA 18:2 (linoleic acid), FA 16:0 (palmitic acid), FA 18:1 (oleic acid) and FA 18:0 (stearic acid) (Fig. 1a), but other less investigated compounds, such as FA 16:4, FA 16:3 or FA 16:2, and oxidized FAs (FA 18:4;O, FA 18:3;O, and FA 18:3;O2) were also identified. Among the 192 compounds identified in ESI (+), the most abundant subclass were TGs (110), followed by oxidized TGs (25), DGs (24), apocarotenoids (6), estolides (4), acylcarnitines (4) and carotenoids (3) (Supplementary Table 2). Figure 1b shows that the most intense peaks correspond to TGs, most of them composed of the FAs identified in ESI (-) mode, such as TG 54:4 | 18:1_18:1_18:2, TG 52:5 | 18:1_18:2_18:2, TG 52:3 | 16:0_18:1_18:2 and TG 54:6 | 18:1_18:2_18:3. In addition to these neutral lipids, 6 apocarotenoids (the cleavage products of carotenoids), three carotenoids (matching the exact mass and elution order of lutein/zeaxanthin, cryptoxanthin and β-carotene), one retinoid (retinal) and one tocopherol (α-tocopherol) were identified. On the other hand, the GC-Q-TOF MS analysis resulted in the annotation of 173 compounds, being FAs and conjugates (9.2%), FAs esters (7.5%), carbohydrates and carbohydrate conjugates (5.8%), carbonyl compounds (5.2%), sesquiterpenoids (4.0%) and monoterpenoids (4.0%) subclasses the most represented (Fig. 1c and Supplementary Table 3). Among them, the most abundant compound was glycerol, followed by several FAs (palmitic acid, docosahexaenoic acid, linoleic acid or linolenic acid), thus confirming the results obtained by CSH-Q/TOF MS/MS-ESI (-). Other interesting compounds were also identified, such as some diterpenoids (phytol, (1 S,3E,7E,11E)-Cembra-3,7,11-triene and geranyl linalool), quinone and hydroquinone lipids (α-tocopherol) and different sesquiterpenoids (beta-ionone, beta-copaene, capsidiol, gamma-elemene, alpha-ionol or (Z)-caryophyllene). In total, more than 350 compounds have been annotated, which contributes to the expansion of the present knowledge on *D. salina* microalgae chemical composition.

### Neuroprotective effect of *Dunaliella salina* extract on Aβ-transgenic *C. elegans* CL4176 paralysis assay

**Fig. 2:**
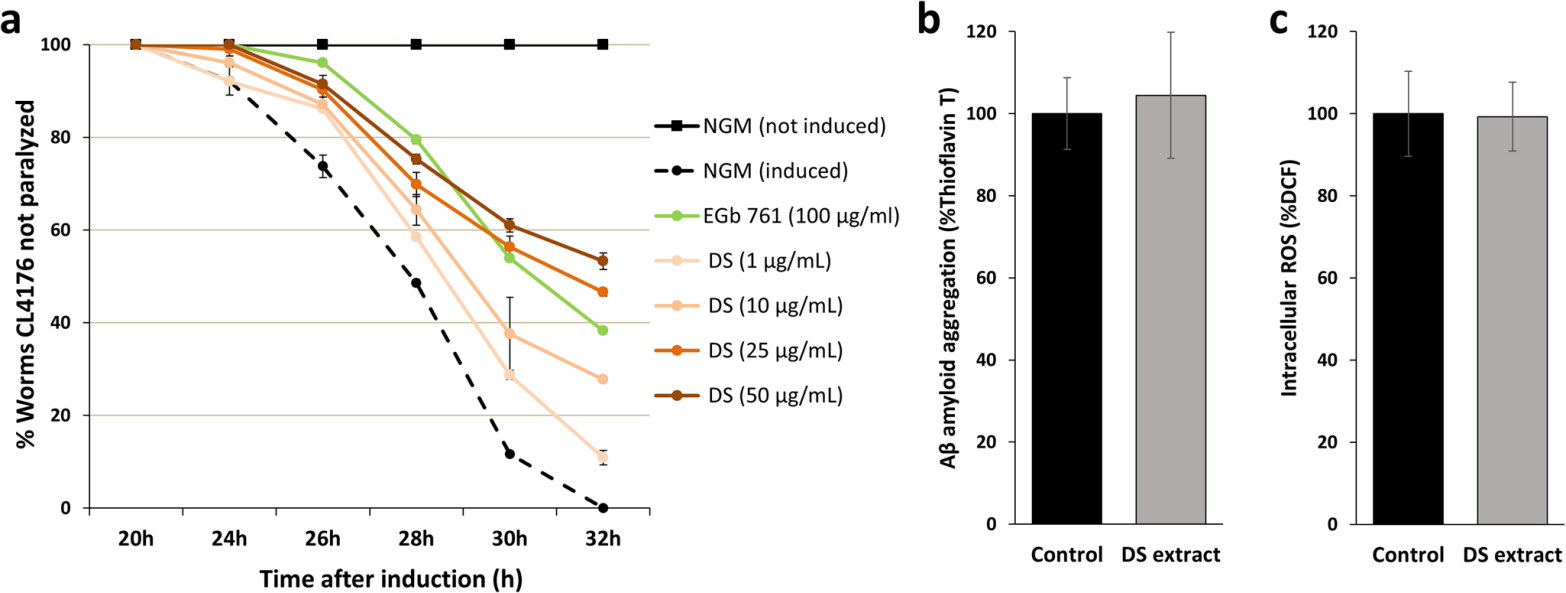
Effect of *Dunaliella salina* (DS) extract on *Caenorhabditis elegans* CL4176 strain. **a** Time course of the paralysis assay after Aβ-peptide induction and treated with different concentrations of DS extract (1, 10, 25 and 50 μg/mL). For each condition tested, two independent assays including *n* = 60 worms/assay were performed. *Ginkgo biloba* extract (EGb 761®) was used as a positive control extract. **b** Percentage of Aβ protein aggregation after 26 h of DS extract treatment (50 μg/mL) compared to control conditions (0.05% DMSO). **c** Percentage of intracellular reactive oxygen species (ROS) after 26 h of DS extract treatment (50 μg/mL) compared to control conditions (0.05% DMSO). In all cases, error bars indicate standard deviation of the mean.

To evaluate the neuroprotective activity of DS extract against the toxicity caused by Aβ1-42 peptide accumulation, the CL4176 nematodes were treated with four doses of the DS extract (1, 10, 25 and 50 μg/mL), and the percentage of worms paralyzed at different times after paralysis induction was determined. The results indicate that there is a dose-dependent protection against paralysis when DS extract was added to the medium and that there is a significant protection against Aβ1-42 peptide toxicity (*p*-value < 0.001) at all doses tested (Fig. 2a). The presence of non-paralyzed worms was also observed in all conditions at the end of the assay (32 h after induction), while the control condition (non-treated with DS extract) showed 100% paralyzed worms. The dose with the lowest number of paralyzed worms during the analysis was 50 μg/mL (53% of not paralyzed worms, *p*-value < 0.0001), with even lower values than the positive control (*Ginkgo biloba* EGb761, 38% of not paralyzed worms, *p*-value < 0.05).

### Transcriptomics analysis in *C. elegans* CL4176

The effect of the DS extract treatment on the transcriptome of the *C. elegans* strain CL4176 was investigated at 26 h after paralysis induction (time at which DS extract protects ≈90% of worms from being paralyzed) using an Illumina RNA-Seq platform. After data processing and quality verification, a total of 19,676 genes were identified, being most of them protein coding RNAs (17,858), pseudogenes (917) and non-coding RNAs (558) (Supplementary Table 4). Then, a differential expression analysis was performed using the DESeq2 package and data were filtered using a 1.5-fold change cut-off threshold (in log2 scale) and an FDR-adjusted *p*-value < 0.05. In total, 150 genes were identified as differentially expressed, of which 120 were up-regulated and 30 were down-regulated. Among these genes, it is interesting to note the high up-regulation of two genes predicted to have aspartic-type endopeptidase activity (*asp-15*, FC = 23.7; *asp-14*, FC = 2.3), two genes involved in the innate immune response (*irg-3*, FC = 11.4; *irg-4*, FC = 3.2), two genes predicted to have oxidoreductase activity (*R05D8.9*, FC = 11.3; *stdh-2*, FC = 9.3), one beta-lactamase domain containing gene (*lact-6*, FC = 11.1), two beta-carotene monooxygenases genes (*bcmo-1*, FC = 8.5; *bcmo-2*, FC = 2.4), and several genes belonging to the cytochrome P450 (CYPs) family or coding for UDP-glucuronosyltransferase (UGTs) proteins. On the other hand, the most down-regulated genes were related to the immune response (*lys-9*, FC = 0.18), the glucosylceramidase activity (*gba-4*, FC = 0.34) or predicted to have carbohydrate binding activity (*clec-50*, FC = 0.48; *clec-71*, FC = 0.51; *clec-60*, FC = 0.52).

**Fig. 3:**
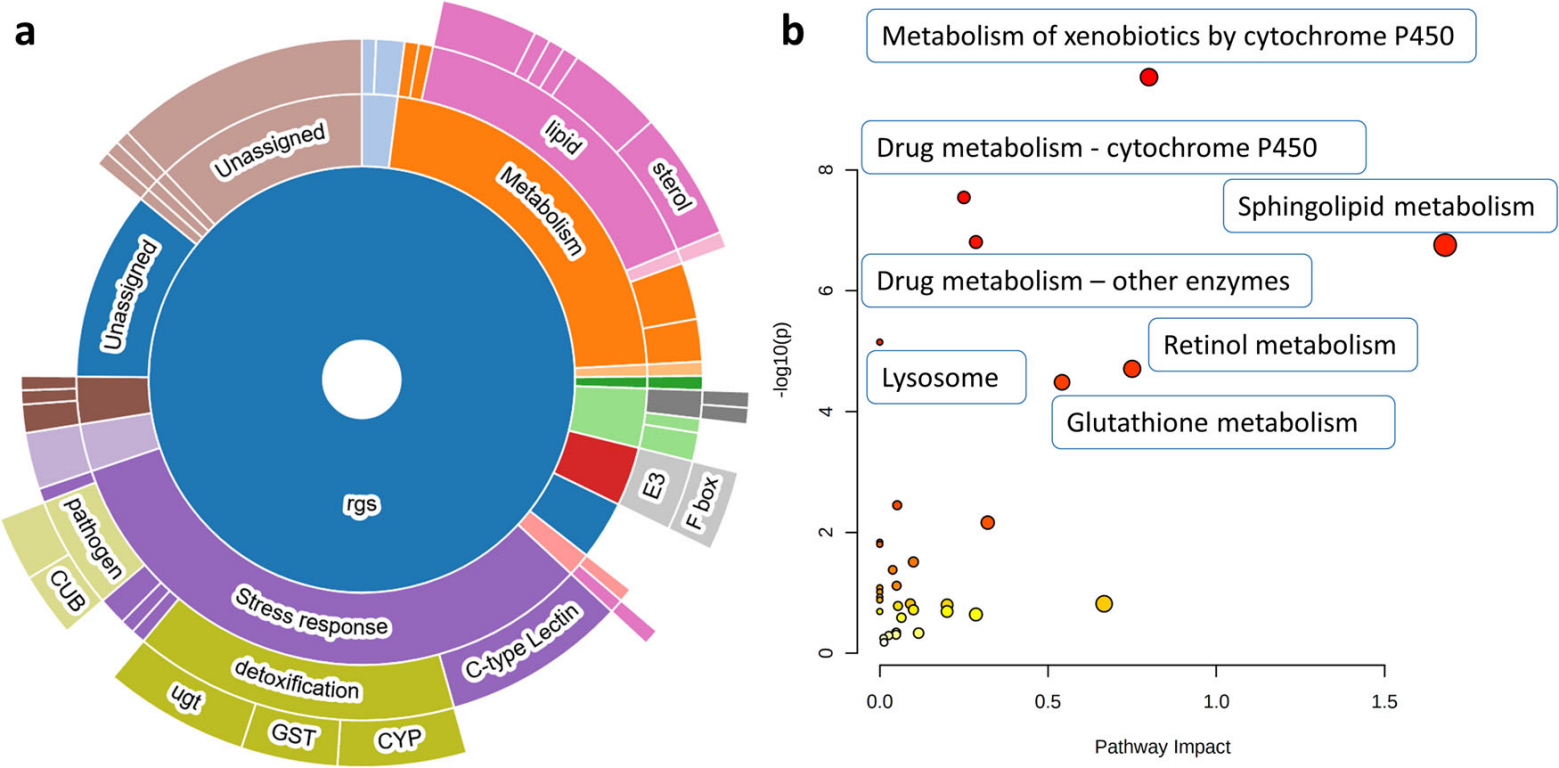
Functional enrichment analysis and pathway analysis of the differentially expressed genes observed in *Caenorhabditis elegans* after *Dunaliella salina* (DS) extract treatment (50 μg/mL, *n* = 5) compared to control conditions (0.05% DMSO, *n* = 5) for 26 h. **a** Sunburst diagram obtained using WormCat 2.0 software. **b** Significantly enriched pathways obtained using MetaboAnalyst.

The generated list of DEG was then explored using the WormCat 2.0 website to identify categories and GO terms significantly enriched after DS extract treatment. As it can be observed in Fig. 3a, the most represented categories were the stress response and the metabolism of lipids. Supplementary Table 5 shows the significantly enriched categories together with their FDR, and the list of genes considered. Among the stress response categories significantly enriched, the CYP detoxification (*cyp-13A10* ↑ , *cyp-14A2* ↑ , *cyp-14A3* ↑ , *cyp-14A4* ↑ , *cyp-25A3* ↑ , *cyp-33C2* ↑ , *cyp-33C4*↑ and *cyp-33E3* ↑ ), the UGT detoxification (*ugt-1* ↑ , *ugt-15* ↑ , *ugt-16* ↑ , *ugt-2* ↑ , *ugt-32* ↑ , *ugt-36* ↑ , *ugt-37* ↑ , *ugt-41*↑ and *ugt-9* ↑ ), the GST detoxification (*gst-10* ↑ , *gst-12* ↑ , *gst-33* ↑ , *gst-39* ↑ , *gst-5*↑ and *gst-6* ↑ ), the C-type Lectin (*clec-143* ↑ , *clec-185* ↑ , *clec-206* ↑ , *clec-210* ↑ , *clec-218* ↑ , *clec-3* ↑ , *clec-70* ↑ , *clec-174* ↓ , *clec-50* ↓ , *clec-60* ↓ , *clec-61* ↓ , *clec-71*↓ and *clec-86* ↓ ) and the response against pathogens (*C32H11.4* ↑ , *F55G11.2* ↑ , *irg-4* ↑ , *dod-24* ↑ , *ilys-2* ↑ , *irg-3* ↑ , *math-38* ↑ , *ZK1037.6*↓ and *kgb-2* ↓ ) were the most relevant. On the other hand, the lipid metabolism was represented by several genes involved in the β-oxidation pathway (*acox-1.1* ↑ , *cpt-5* ↑ , *F53C11.3* ↑ , *Y87G2A.2* ↑ , *cpt-3*↓ and *W03D8.8* ↓ ), the sterol metabolism (*C06E4.3* ↑ , *D1054.8* ↑ , *F12E12.11* ↑ , *F12E12.12* ↑ , *F25D1.5* ↑ , *R05D8.7* ↑ , *R05D8.9*↑ and *stdh-2* ↑ ) and the sphingolipid metabolism (*asah-1* ↑ , *asm-2* ↑ , *asm-3* ↑ , *gba-2* ↑ , *gba-4*↓ and *spl-2* ↓ ).

The complementary enrichment analysis performed using WormExp revealed significant over-represented gene sets related to different transcription factor genes, such as ELT-2^35–37^, PQM-1^38^, PMK-1^39^, SKN-1^40,41^, DAF-16^42^, or NPR-1^43^ (Table 1). Moreover, the pathway enrichment analysis performed by MetaboAnalyst 5.0 (Fig. 3b) showed three interconnected pathways significantly enriched (with all the genes up-regulated), such as the *Metabolism of xenobiotics and drugs mediated by cytochrome P450* (*gst-6*, *gst-4*, *gst-5*, *ugt-15*, *D2063.1*, *sodh-2*, *gst-10* and *W01A11.1*) and the *Drug metabolism mediated by other enzymes* (*C52A10.2*, *F13H6.3*, *ugt-15*, *gst-6*, *gst-4*, *gst-5* and *gst-10*). Other enriched pathways were the *Sphingolipid metabolism*, with 4 genes up-regulated (*asah-1*, *asm-3*, *asm-2*, *gba-2*) and two genes down-regulated (*gba-4* and *spl-2*), the *Lysosome metabolism* (*ctsa-1.2*, *gba-2*, *lipl-1*, *asm-3*, *asm-2*, *asah-1* and *gba-4*), the *Retinol metabolism* (with 5 upregulated genes: *D2063.1*, *sodh-2*, *dhrs-4* and *ugt-15*) and the *Glutathione metabolism* (with all genes upregulated: *gpx-1*, *gst-6*, *gst-4*, *gst-5* and *gst-10*) (Supplementary Table 6). It is interesting to note that the KEGG database does not consider *bcmo-1* and *bcmo-2* genes (highly up-regulated by DS extract) in the *Retinol metabolism*, but they are considered by WormPaths, a web-based collection of standardized metabolic pathway maps for *C. elegans* (http://wormflux.umassmed.edu/), and therefore they were considered part of the retinol pathway.

**Table 1:** Enriched WormExp transcription factors gene sets using the 150 differentially expressed genes after the treatment with DS extract at 50 μg/mL (*n* = 4) in comparison to control conditions (*n* = 4) after 26 h.

Transcription factor gene set	Counts	Bonferroni	Overlapped genes	Reference
Down in wt vs elt-2(-);elt-7(-) larvae	40	7.33E-22	irg-3 ↑ , ugt-15 ↑ , T16G1.7 ↑ , clec-210 ↑ , F42A10.7 ↑ , C32H11.4 ↑ , asm-3 ↑ , irg-4 ↑ , T16G1.4 ↑ , lipl-1 ↑ , clec-218 ↑ , ugt-16 ↑ , clec-3 ↑ , asm-2 ↑ , asah-1 ↑ , hsp-12.3 ↑ , asp-14 ↑ , F47H4.2 ↑ , math-38 ↑ , cav-2 ↑ , ilys-2 ↑ , Y57G11C.41 ↑ , pcp-1 ↑ , F42A10.6 ↑ , acds-10 ↑ , clec-143 ↑ , dod-24 ↑ , C32D5.6 ↑ , F13D12.6 ↑ , Y87G2A.2 ↑ , kgb-2 ↓ , W03D8.8 ↓ , ZK896.5 ↓ , clec-61 ↓ , clec-86 ↓ , scav-5 ↓ , spp-17 ↓ , clec-50 ↓ , gba-4 ↓ , C17F4.7↓	^37^
PQM-1 L3 Targets	49	1.22E-21	irg-3 ↑ , ugt-15 ↑ , T16G1.7 ↑ , F25D1.5 ↑ , T16G1.6 ↑ , F42A10.7 ↑ , C32H11.4 ↑ , irg-4 ↑ , T16G1.4 ↑ , gst-33 ↑ , clec-218 ↑ , ugt-16 ↑ , asm-2 ↑ , Y32B12C.1 ↑ , asah-1 ↑ , F47H4.2 ↑ , ugt-32 ↑ , EGAP9.3 ↑ , cav-2 ↑ , dhrs-4 ↑ , gba-2 ↑ , ugt-37 ↑ , ilys-2 ↑ , F07C3.9 ↑ , gst-6 ↑ , gst-4 ↑ , pcp-1 ↑ , C31C9.7 ↑ , F10C2.3 ↑ , acds-10 ↑ , ugt-2 ↑ , pgp-1 ↑ , pgp-9 ↑ , acox-1.1 ↑ , F13D12.6 ↑ , F53C11.3 ↑ , K11H12.4 ↑ , F13H6.3 ↑ , lec-11 ↑ , ndnf-1 ↓ , ZK896.5 ↓ , clec-86 ↓ , scav-5 ↓ , clec-60 ↓ , Y51F10.7 ↓ , spp-17 ↓ , clec-50 ↓ , gba-4 ↓ , C17F4.7↓	^38^
Down in wt vs elt-2(-) larvae	29	1.32E-16	irg-3 ↑ , R09E12.9 ↑ , C32H11.4 ↑ , asm-3 ↑ , irg-4 ↑ , T16G1.4 ↑ , clec-218 ↑ , clec-3 ↑ , F55G11.2 ↑ , asp-14 ↑ , cyp-14A2 ↑ , math-38 ↑ , cav-2 ↑ , ugt-37 ↑ , Y57G11C.41 ↑ , gst-6 ↑ , F42A10.6 ↑ , acds-10 ↑ , clec-143 ↑ , cyp-13A10 ↑ , F46C5.10 ↑ , dod-24 ↑ , gst-10 ↑ , kgb-2 ↓ , clec-86 ↓ , scav-5 ↓ , spp-17 ↓ , clec-50 ↓ , gba-4↓	^37^
PMK-1 targets down in Day 15 vs. Day 6	23	3.12E-15	irg-3 ↑ , clec-210 ↑ , F25D1.5 ↑ , F42A10.7 ↑ , asm-3 ↑ , irg-4 ↑ , lipl-1 ↑ , asm-2 ↑ , asp-14 ↑ , cpt-5 ↑ , F01D5.2 ↑ , pcp-1 ↑ , C36C5.5 ↑ , F42A10.6 ↑ , dod-24 ↑ , F13D12.6 ↑ , gst-10 ↑ , D1054.8 ↑ , clec-86 ↓ , clec-60 ↓ , Y51F10.7 ↓ , clec-50 ↓ , gba-4↓	^39^
Low-complexity elt-2 targets	48	3.97E-14	irg-3 ↑ , lact-6 ↑ , ugt-15 ↑ , T16G1.7 ↑ , clec-210 ↑ , T16G1.6 ↑ , F42A10.7 ↑ , C32H11.4 ↑ , irg-4 ↑ , T16G1.4 ↑ , lipl-1 ↑ , clec-218 ↑ , ugt-16 ↑ , F55G11.2 ↑ , asm-2 ↑ , bcmo-2 ↑ , asah-1 ↑ , gst-5 ↑ , asp-14 ↑ , math-38 ↑ , ugt-32 ↑ , gba-2 ↑ , ilys-2 ↑ , F07C3.9 ↑ , pcp-1 ↑ , F42A10.6 ↑ , ugt-2 ↑ , clec-143 ↑ , pgp-1 ↑ , pgp-9 ↑ , acox-1.1 ↑ , M01A8.1 ↑ , F13D12.6 ↑ , lec-11 ↑ , ZK1037.6 ↓ , mfb-1 ↓ , W03D8.8 ↓ , clec-174 ↓ , F45D11.1 ↓ , ZK896.5 ↓ , clec-61 ↓ , clec-86 ↓ , scav-5 ↓ , spl-2 ↓ , clec-60 ↓ , Y51F10.7 ↓ , clec-50 ↓ , C17F4.7↓	^35^
Down in elt-2(-) larvae vs elt-2(-);elt-7(-) larvae	24	4.12E-11	ugt-15 ↑ , lbp-8 ↑ , clec-210 ↑ , F42A10.7 ↑ , C32H11.4 ↑ , ugt-1 ↑ , lipl-1 ↑ , ugt-16 ↑ , asm-2 ↑ , Y32B12C.1 ↑ , F47H4.2 ↑ , pcp-1 ↑ , F42A10.6 ↑ , F13D12.6 ↑ , K11H12.4 ↑ , pals-6 ↓ , ZK1037.6 ↓ , clec-174 ↓ , ZK896.5 ↓ , clec-61 ↓ , B0563.9 ↓ , clec-50 ↓ , Y47H10A.5 ↓ , C17F4.7↓	^37^
skn-1 targets (positive regulation)	10	2.58E-09	cyp-14A4 ↑ , gst-14 ↑ , gst-5 ↑ , cyp-14A2 ↑ , gst-12 ↑ , gst-6 ↑ , gst-4 ↑ , gst-10 ↑ , F56D5.3 ↑ , gst-39↑	^40^
daf-16 targets	24	4.59E-09	R09E12.9 ↑ , F25D1.5 ↑ , C32H11.4 ↑ , F12E12.11 ↑ , gst-33 ↑ , bcmo-2 ↑ , hsp-12.3 ↑ , C06E4.3 ↑ , cav-2 ↑ , cdr-4 ↑ , pcp-1 ↑ , F10C2.3 ↑ , F46C5.10 ↑ , F53C11.3 ↑ , D2063.1 ↑ , C03G6.5 ↑ , F13H6.3 ↑ , ugt-41 ↑ , Y87G2A.2 ↑ , scav-5 ↓ , clec-60 ↓ , Y51F10.7 ↓ , clec-50 ↓ , C17F4.7↓	^42^
14 skn-1 targets	7	1.16E-08	C32H11.4 ↑ , F55G11.2 ↑ , asp-14 ↑ , gst-4 ↑ , dod-24 ↑ , gst-10 ↑ , F56D5.3↑	^41^
daf-16 targets within daf-2(-)	19	5.03E-05	R09E12.9 ↑ , dhs-23 ↑ , F25D1.5 ↑ , F12E12.11 ↑ , gst-33 ↑ , bcmo-2 ↑ , hsp-12.3 ↑ , cav-2 ↑ , cdr-4 ↑ , F46C5.10 ↑ , F53C11.3 ↑ , D2063.1 ↑ , F56D5.3 ↑ , C03G6.5 ↑ , F13H6.3 ↑ , ugt-41 ↑ , Y87G2A.2 ↑ , mfb-1 ↓ , clec-60↓	^42^
elt-2 targets	9	2.72E-04	ugt-16 ↑ , cav-2 ↑ , gba-2 ↑ , pcp-1 ↑ , acox-1.1 ↑ , W03D8.8 ↓ , Y51F10.7 ↓ , clec-50 ↓ , C17F4.7↓	^36^
Age-regulated elt-2 targets	10	3.05E-03	irg-3 ↑ , F42A10.7 ↑ , C32H11.4 ↑ , irg-4 ↑ , lipl-1 ↑ , asm-2 ↑ , asp-14 ↑ , pcp-1 ↑ , F42A10.6 ↑ , F13D12.6↑	^35^
Up in wt vs elt-2(-) larvae	19	4.55E-03	cyp-14A4 ↑ , spp-9 ↑ , lbp-8 ↑ , ugt-1 ↑ , Y32B12C.1 ↑ , cdr-4 ↑ , F07C3.9 ↑ , F10C2.3 ↑ , pgp-1 ↑ , K11H12.4 ↑ , lec-11 ↑ , pals-6 ↓ , ZK1037.6 ↓ , clec-174 ↓ , ndnf-1 ↓ , Y51F10.7 ↓ , B0563.9 ↓ , Y47H10A.5 ↓ , dod-23↓	^37^
npr-1 associated by eQTL	9	7.08E-03	C36C5.15 ↑ , C36C5.14 ↑ , cyp-14A2 ↑ , F01D5.2 ↑ , K09C6.9 ↑ , C36C5.5 ↑ , K11H12.4 ↑ , D2063.1 ↑ , spp-17↓	^43^

Significance was determined using Bonferroni FDR < 0.05.

Among the lists of DEGs by DS extract, four genes with remarkable expression ratios, significance level and biological importance were selected for RT-qPCR validation: *bcmo-1*, involved in the metabolism of carotenoids; *asm-3* and *lipl-1* involved in the metabolism of lipids; and *gst-14*, involved in the oxidative stress response (and controlled by SKN-1 transcription factor). As shown in Table 2, the expression ratio of these genes was statistically significant (*p*-value < 0.05), confirming the results obtained by RNA-Seq (with a Pearson correlation *r* value of 0.967 between RNA-Seq and RT-qPCR values).

**Table 2:** Comparison of gene expression ratios in *C. elegans* in response to DS extract treatment at 50 μg/mL (*n* = 4) in comparison to control conditions (*n* = 4) after 26 h, and determined by RNA-Seq and RT-qPCR techniques.

Gene symbol (Accession N.)	RNA-Seq		RT-qPCR	
	FC^a^	*p-value*^*b*^	FC^a^	*p-value*^*c*^
*bcmo-1* (Y46G5A.24)	8.48	1.54*10^−7^	6.34	0.024
*asm-3* (W03G1.7)	3.40	5.65*10^−4^	3.22	0.026
*lipl-1* (F54F3.3)	2.91	1.09*10^−4^	3.63	0.027
*gst-14* (F37B1.3)	3.55	2.64*10^−9^	4.12	0.003
Aβ	-	-	0.71	0.093

^a^Fold change (expression ratio).

^b^Adjusted *p*-value (FDR).

^c^Statistical significance calculated by REST2009.

### Effect of *Dunaliella salina* extract on Aβ gene expression, Aβ protein aggregation and intracellular reactive oxidative species (ROS) levels in *C. elegans* CL4176

Apart from the validation of RNA-Seq results, RT-qPCR was applied to evaluate the possible direct effect of DS extract on the expression of Aβ gene at the transcript level 26 h after paralysis induction. The results obtained from four independent experiments showed that DS extract slightly reduced the Aβ gene expression compared to the control samples (FC = 0.71), but this change was not statistically significant (*p*-value = 0.093) (Table 2). Complementary, the detection of intracellular Aβ amyloid protein aggregates was evaluated by the use of thioflavin T stain. Our results demonstrated that the levels of Aβ aggregates was not affected by DS extract (99.3%, *p*-value = 0.908) (Fig. 2b), therefore discarding the direct effect of DS extract on Aβ amyloid gene expression or protein aggregation. Furthermore, *C. elegans* intracellular levels of ROS were evaluated by using the 2′,7′-dichlorofluorescein diacetate (DCF-DA) fluorescent probe. Our data showed that DS extract had no effect on the intracellular ROS levels in treated worms compared to control worms (104.5%, *p*-value = 0.596) (Fig. 2c). This result suggests that the direct antioxidant activity is not one of the factors contributing to the in vivo neuroprotective activity of DS extract.

### Metabolomics analysis in *C. elegans* CL4176

**Fig. 4:**
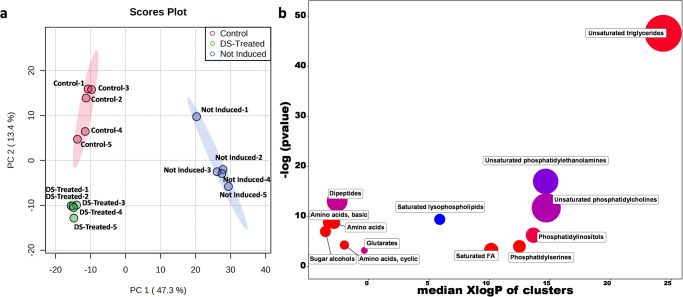
Multivariate analysis and ChemRICH results of the metabolomics data from *Caenorhabditis elegans* experiments. **a** Principal Component Analysis score plots (PC1 vs PC2) including the three groups of analyzed samples (Control, DS-Treated, Not Induced) (*n* = 5 for each group). **b** Chemical similarity enrichment results obtained from DS-Treated samples (50 μg/mL, *n* = 5) compared to Control conditions (0.05% DMSO, *n* = 5) for 26 h. The *y*-axis shows the most significantly altered clusters on top; the *x*-axis shows the XlogP values of clusters. Cluster colors give the proportion of increased or decreased compounds (red = increased, blue = decreased) in each cluster. Chemical enrichment statistics is calculated by Kolmogorov-Smirnov test. Only enrichment clusters are shown that are significantly different at *p* < 0.05.

To yield a comprehensive view of the intracellular metabolic changes performed by DS extract treatment, an untargeted metabolomic analysis on *C. elegans* was performed. For this aim, three groups of worms were included: one cultured at 25 °C in NGM (+DMSO 0.05%) and termed “Control”; one cultured at 25 °C in NGM (+50 μg/mL DS extract) and termed “DS-Treated”; and a third group cultured at 16 °C in NGM (+DMSO 0.05%) and termed “Not Induced”. To expand the coverage of identify metabolites, three different analytical platforms (CSH-Q-TOF MS/MS for lipidomics; HILIC-Q-TOF MS/MS and GC-Q-TOF MS for primary metabolism analysis) were applied. Data obtained from each analytical platform (and ionization modes) were processed independently. The relative standard deviation of the internal standards included during sample preparation and analysis is shown in Supplementary Table 7, and the list of annotated compounds for each analytical platform can be found in Supplementary Tables 8–12. After data processing, the lipidomics analysis resulted in the annotation 430 lipids in ESI (+), 197 lipids in ESI (-), 146 metabolites in HILIC-ESI (+), 77 metabolites in HILIC-ESI (-), and 94 metabolites in GC-MS. All these data were combined, duplicate metabolites were removed and a joined dataset was generated, which resulted in the annotation of 793 compounds (Supplementary Table 13). The PCA of this joined dataset demonstrates that the three groups of samples (“Control”, “DS-Treated”, and “Not Induced”) are clearly separated (Fig. 4a). It also shows that “DS-Treated” samples are closer to the “Control” samples, and these two groups are markedly separated from the “Not Induced” samples. Furthermore, the univariate ANOVA analysis shows 573 metabolites as significantly different between the three groups of samples (FDR < 0.05), most of them occurring between the Aβ1-42 induced samples (“Control” and “DS-Treated”) and the “Not Induced” samples, suggesting Aβ1-42 peptide accumulation through temperature up-shift as the main difference between the analyzed groups.

### Comparison between control and not induced conditions

To get a deeper knowledge on the effects of the induction of Aβ1-42 peptide accumulation, a *T*-Test univariate analysis was performed between the “Control” and the “Not Induced” groups. Setting a FC threshold of 1.5 and an FDR < 0.05, almost 50% of the metabolites were significantly altered (368), most of them with higher abundance after Aβ1-42 peptide accumulation (335), and some of them with lower abundance (33) (Supplementary Table 13). To analyze the chemical diversity represented in these metabolites, a chemical enrichment analysis using ChemRICH was performed (Supplementary Fig. 1). As expected due to the high number of significantly increased metabolites, many chemical classes were significantly increased, such as unsaturated TGs, unsaturated PCs, unsaturated PEs, saturated lysophospholipids, unsaturated LPCs, saturated TGs, saturated LPCs and saturated PCs. Other chemical classes (dipeptides, unsaturated ceramides and saturated FAs) were also altered, with some species increased, others decreased. On the other hand, purines was the only chemical class significantly decreased. The list of altered metabolites was also analyzed by MetaboAnalyst 5.0 to identify overrepresented biological pathways (out of the 368 altered metabolites, only 84 had HMDB IDs and 47 had KEGG IDs, and most lipids could not be mapped). This analysis showed the *Valine, Leucine and Isoleucine biosynthesis* pathway as the most significantly enriched, with 3 increased metabolites matching to this pathway (3-methyl-2-oxovaleric acid, 4-methyl-2-oxovaleric acid and L-threonine), followed by the *Arginine biosynthesis* pathway, with 2 metabolites (ornithine and glutamine), and the *Purine metabolism* pathway, with three increased metabolites (glutamine, guanine and guanosine) and two decreased metabolites (inosine and uric acid).

### Comparison between DS-treated and control conditions

Finally, and to understand the DS extract treatment effects on the metabolism of *C. elegans* and how these changes could be related to its neuroprotective effect, a comparison between the “DS-Treated” group and the “Control” group was performed. In this comparison, 69 metabolites were significantly altered, 52 with higher abundance and 17 with lower abundance (Table 3). The chemical enrichment analysis performed by ChemRICH also showed unsaturated TGs, amino acids, sugar alcohols, phosphatidylinositols, phosphatidylserines and saturated FA as significantly increased. In addition, a few chemical classes were altered with some species increased, others decreased, such as dipeptides, unsaturated PCs and glutarates (Fig. 4b). Unsaturated PEs and saturated lysophospholipids were markedly decreased. The metabolite pathway analysis showed the *Valine, Leucine and Isoleucine biosynthesis* pathway as the most significantly enriched pathway, with 2 decreased metabolites (4-methyl-2-oxovaleric acid and 3-methyl-2-oxovaleric acid). The second most enriched pathway was the *Glutathione metabolism*, considering cadaverine (with decreased values) and ornithine (with increased values) after DS extract treatment.

**Table 3:** Altered metabolites in *C. elegans* after DS extract treatment at 50 μg/mL (*n* = 5) in comparison to control conditions (*n* = 5) after 26 h.

*Metabolite name*	*Analytical Platform*	*MSI level*	*FC*	*FDR*	*Chemical class*
Ala-Ala	HILIC (+)	2a	2.76	0.0026	Dipeptides
6-Methyladenosine	HILIC (+)	2b	2.45	0.0017	Purine nucleosides
N-Methylhistidine	HILIC (+)	2a	2.43	0.0036	Histidine derivatives
Targinine	HILIC (+)	2a	2.41	0.0020	Arginine derivatives
Proline-hydroxyproline	HILIC (+)	2a	2.40	0.0009	Dipeptides
PC p-36:1/PC o-36:2 A	CSH (+)	1	2.36	0.0015	Unsaturated PC
PC 39:9	CSH (+)	2b	2.36	0.0007	Unsaturated PC
Glu-Thr	HILIC (+)	2a	2.11	0.0017	Dipeptides
3-Methylhistidine	HILIC (+)	2a	2.08	0.0449	Histidine derivatives
PC 37:6 A	CSH (+)	2b	2.03	0.0007	Unsaturated PC
PC 39:8	CSH (+)	2b	2.02	0.0027	Unsaturated PC
Inosine	GCMS	1	2.01	0.0424	Purine nucleosides
3′-O-Methylguanosine	HILIC (+)	2a	1.98	0.0143	Purine nucleosides
gamma-Glu-Gln	HILIC (+)	2a	1.96	0.0449	Dipeptides
PE O-37:5	CSH (+)	2a	1.95	0.0013	Ether-linked PE
PC 39:10 | PC 19:5_20:5	CSH (-)	2b	1.95	0.0015	Unsaturated PC
DL-o-Tyrosine	HILIC (+)	2b	1.92	0.0316	Phenylalanine derivatives
FA 19:3	CSH (-)	2b	1.92	0.0320	Unsaturated FA
Arabitol	GCMS	1	1.89	0.0014	Sugar alcohol
3′-O-Methylcytidine	HILIC (+)	2a	1.89	0.0107	Pyrimidine nucleosides
FA 19:5	CSH (-)	2b	1.87	0.0322	Unsaturated FA
2-Hydroxyglutaric acid	GCMS	1	1.84	0.0089	Glutarates
PI 37:5	CSH (+)	2b	1.83	0.0017	PI
Lactic acid	GCMS	1	1.80	0.0026	Lactates
PC 37:7	CSH (+)	2b	1.79	0.0013	Unsaturated PC
PI 39:5	CSH (+)	2b	1.79	0.0055	PI
FA 19:2	CSH (-)	2b	1.78	0.0235	Unsaturated FA
N,N-Dimethylarginine	HILIC (+)	1	1.77	0.0007	Arginine derivatives
PE P-37:5 | PE P-17:0_20:5	CSH (+)	2b	1.77	0.0013	Ether-linked PE
FA 19:4	CSH (-)	2b	1.76	0.0428	Unsaturated FA
NAE 19:3	CSH (+)	2b	1.73	0.0404	N-acylethanolamines
PE P-37:1 | PE P-19:0_18:1	CSH (+)	2b	1.71	0.0017	Ether-linked PE
PI 37:5 | PI 17:0_20:5	CSH (-)	2b	1.69	0.0017	PI
Glucuronic acid	HILIC (-)	2a	1.69	0.0069	Glucuronic acid derivatives
N-Acetylserine	HILIC (-)	2a	1.68	0.0358	amino acids, modified
TG 53:5	CSH (+)	2b	1.63	0.0240	Unsaturated TG
LPI 20:5	HILIC (-)	2b	1.62	0.0406	Phosphatidylinositols
TG 55:9 | TG 18:2_18:2_19:5	CSH (+)	1	1.61	0.0030	Unsaturated TG
TG 56:9 | TG 18:1_18:3_20:5	CSH (+)	2b	1.60	0.0056	Unsaturated TG
TG 58:10 | TG 18:1_20:4_20:5	CSH (+)	1	1.59	0.0026	Unsaturated TG
Galacturonic acid	GCMS	1	1.59	0.0013	Glucuronic acid derivatives
TG 57:8 | TG 17:1_20:3_20:4	CSH (+)	2a	1.59	0.0007	Unsaturated TG
Dehydroascorbic acid	GCMS	1	1.58	0.0197	Gamma butyrolactones
TG 60:14 | TG 20:4_20:5_20:5	CSH (+)	2b	1.58	0.0015	Unsaturated TG
Hypoxanthine	HILIC (-)	1	1.58	0.0030	Hypoxanthines
TG 54:7	CSH (+)	1	1.57	0.0076	Unsaturated TG
TG 58:11 | TG 18:3_20:3_20:5	CSH (+)	1	1.55	0.0078	Unsaturated TG
TG 58:8 | TG 18:1_20:3_20:4	CSH (+)	2b	1.54	0.0007	Unsaturated TG
Ornithine	GCMS	1	1.53	0.0026	L-alpha-amino acids
Glycerophosphocholine	HILIC (+)	2b	1.52	0.0014	Glycerophosphocholines
TG 58:13 | TG 18:3_20:5_20:5	CSH (+)	2b	1.51	0.0069	Unsaturated TG
alpha-Galactosamine-1-phosphate	HILIC (+)	2a	1.51	0.0322	Monosaccharide phosphates
Orotic acid	HILIC (-)	2a	0.14	0.0197	Pyrimidinecarboxylic acids
PMeOH 40:10|PMeOH 20:5_20:5	CSH (-)	2b	0.35	0.0475	Glycerophospholipids
3-Methyl-2-oxovaleric acid	HILIC (-)	2a	0.43	0.0192	keto acids
4-Methyl-2-oxovaleric Acid	HILIC (-)	2a	0.45	0.0132	keto acids
Cadaverine	HILIC (+)	2a	0.46	0.0445	biogenic polyamines
LPE 19:1	CSH (-)	2b	0.46	0.0024	Unsaturated LPE
Lys-Pro	HILIC (+)	2b	0.53	0.0272	Dipeptides
LPE 18:0	CSH (-)	2b	0.61	0.0011	Saturated LPE
Ala-Leu	HILIC (+)	2a	0.62	0.0206	Dipeptides
Methylphosphate	GCMS	1	0.63	0.0235	Organophosphates
PE 39:6	CSH (+)	2b	0.64	0.0035	Unsaturated PE
TG 56:3 | TG 18:1_19:1_19:1	CSH (+)	2a	0.65	0.0179	Unsaturated TG
TG 55:2 | TG 18:0_18:0_19:2	CSH (+)	2a	0.66	0.0318	Unsaturated TG
PI 39:6	CSH (+)	2b	0.66	0.0090	PI
LPE 15:0	CSH (-)	2b	0.66	0.0318	Saturated LPE
PE 33:3 | PE 15:0_18:3	CSH (-)	2b	0.66	0.0251	Unsaturated PE
PE 37:2 | PE 18:1_19:1	CSH (-)	2b	0.67	0.0127	Unsaturated PE

Significance was determined using a FC threshold (≥1.5 for increased metabolites and ≤0.67 for decreased metabolites), and a *T*-Test with FDR < 0.05.

*MSI* Metabolomics Standards Initiative: 1, *m/z*, MS/MS, RT; 2a, *m/z*, RT; 2b, *m/z*, MS/MS. *FC* fold change, *FDR* false discovery rate.

*PC* phosphatidylcholine, *PE* phosphatidylethanolamine, *FA* fatty acid, *PI* phosphatidylinositol, *NAE* N-acylethanolamine, *TG* triacylglycerol, *LPI* lysophosphatidylinositol, *LPE* lysophosphatidylethanolamine.

## Discussion

Previous studies from our laboratory have reported the carotenoid composition of the DS extract used in the present study, being all-trans-β-carotene the most abundant compound, and followed by lutein and all-trans-α-carotene^44^. That study highlighted that carotenoids only represented 5.2% (w/w) of the total composition of the DS extract, whereas the remainder of the extract could be other relatively non-polar components such as glycerol and other lipids^45^. The FAs profile of different *D. salina* extracts has also been previously determined by our research group and others, demonstrating the high abundance of palmitic (FA 16:0), linolenic (FA 18:3), palmitoleic (FA 16:1), docosahexaenoic (FA 22:6) and oleic (FA 18:1) acids^46–48^, some of which are essential and of great importance for the brain development^49^. In addition to carotenoids, FAs, polar lipids, glycerol, proteins and carbohydrates, *D. salina* also contains a large diversity of sterols^50^, and other volatiles compounds related to carotenoid degradation (the monoterpene β-cyclocitral and the sesquiterpenes α- and β-ionone) or diterpenoids, such as phytol^46^. The results obtained in the present study confirm the presence of 3 carotenoids previously reported, but due to the limitations of the chromatographic separation and the ionization mode used (ESI), the different isomers could not be distinguished. In addition, 6 apocarotenoids resulting from the oxidative cleavage of carotenoids were identified, many of them highly abundant in DS extract, such 10’-apo-β-carotenal, 14’-apo-β-carotenal, 13-Apo-β-carotenone and 12’-apo-β-carotenal. Our results also confirm the presence of 44 FAs, being the most abundant those already described in the literature^46–48^. However, other FAs not previously reported have been identified, such as oxidized FAs (FA 18:4;O, FA 18:3;O, and FA 18:3;O2). Furthermore, the most abundant FAs are forming part of more complex lipids, such as TGs, DGs and oxidized TGs, and also the presence of two acylhexosyl campesterols (ASG 28:1;O;Hex;FA 16:0 and ASG 28:1;O;Hex;FA 18:1) and 4 TG-estolides [TG 64:1;O2 | TG 16:0_16:0_16:0;O(FA 16:0), TG 66:2;O2 | TG 16:0_18:1_16:0;O(FA 16:0), TG 66:3;O2 | TG 16:0_16:0_18:1;O(FA 16:1), TG 66:8;O2 | TG 16:1_18:3_16:2;O(FA 16:1)]. We have also confirmed the high abundance of glycerol, and the presence of different diterpenoids, several sesquiterpenoids and α-tocopherol, thus increasing the knowledge on the chemical composition of *D. salina*. However, it has to be noticed that actual databases do not contain extensive information about microalgal compounds and therefore, some of the compounds identified by MS or MS/MS spectral similarity match might or might not be totally accurate, and this information must be confirmed in future studies^51^.

The unique and complex chemical composition of *D. salina* makes this microalga a potential source of neuroprotective compounds that could interfere with one or more AD hallmarks. Carotenoids have shown to protect the cellular components against ROS^14^, enhance the endogenous antioxidant systems^17^, and modulate inflammation-related mechanisms^15^, and these effects have been demonstrated also in vivo using *C. elegans* models^24–29^. Apart from carotenoids and their antioxidant capacity, transgenic *C. elegans* strains have also been used to evaluate the protection of different natural extracts against the toxicity of Aβ plaques formation^52^. In the present study, the *C. elegans* paralysis assay demonstrated that the DS extract at 50 and 25 μg/mL (Fig. 2a) has a better neuroprotective activity than the commercial *Ginkgo biloba* EGb761® extract (used as positive control). This activity agrees well with a previous work of our research group where we have demonstrated its neuroprotective activity against Aβ1-42 toxic effects in neuroblastoma cells^18^. In that work we also confirmed the antioxidant capacity of DS extract against the massive oxidative stress induced by L-glutamic acid, its anti-inflammatory capacity using a lipoxygenase (LOX) enzymatic assay, and the inhibition of pro-inflammatory cytokine release in the THP-1 monocyte cell culture model. These effects could be related to the high carotenoids content of DS extract (β-carotene, lutein and zeaxanthin), whose implication as natural antioxidant and anti-neuroinflammation agents has been already demonstrated in vitro and in vivo^53^. However, the results of the present study demonstrated that the neuroprotective activity of DS in *C. elegans* is not related to the reduction of the intracellular ROS levels, the inhibition of Aβ gene expression or the decrease of Aβ protein aggregation. Similar results have been previously observed for some *Lycoris radiata* compounds, which can inhibit the paralysis of CL4176 worms after temperature up-shift without having antioxidant activity, nor reducing the Aβ gene expression at both the transcript and peptide levels^54^. The authors of that work suggested that the anti-paralysis effects of *Lycoris* compounds is mainly due to their AChE gene inhibition and inflammation/stress-related gene modulation, which is partly compatible to our present results.

Other possible mechanisms involved in the reduction of *C. elegans* paralysis by DS extract might be the regulation of different transcription factors. For instance, *Citrus sinensis* extracts enriched in carotenoids have shown to alleviate the paralysis induced by Aβ toxicity^28,29^. In these studies, the authors demonstrated that orange juices up-regulate the expression of detoxification and antioxidant response genes (*gcs-1*, *gst-4*, *sod-3*, *hsp-4* or *hsp-16.2*) by acting through the transcription factor SKN-1, but also delaying the Aβ-amyloid-induced paralysis in a manner requiring SKN-1, DAF-16, and HSF-1 transcription factors. The SKN-1 transcription factor is the homolog of the mammalian NRF2, and it activates the detoxification response and the resistance to oxidative stress^40,55^. However, SKN-1 must be phosphorylated by the map kinase (MAPK) pathway through a signaling cascade to be fully activated^56^. Based on the transcription factor enrichment analysis performed by WormExp, our transcriptomics data suggest that DS extract treatment activates SKN-1, as the expression of several genes controlled by this transcription factor is increased (Supplementary Table 4). The activation of SKN-1 would be responsible of the enrichment of several stress response categories and metabolic pathways (CYP, UGT and GST mediated detoxification processes), which might be connected to the protection observed in the paralysis assay. However, since the *skn-1* gene expression was not affected in our study (FC = 0.96; FDR = 0.999), future experiments knocking down *skn-1* will be performed to confirm this hypothesis. GSTs, UGTs and CYPs are also important metabolic enzymes in various tissues, including brain, where they can mediate the synthesis and metabolism of some endogenous and other substances. Moreover, CYPs have been involved in the metabolism of some bioactive secondary metabolites including carotenoids and apocarotenoids^57,58^, which is directly connected with the activation of the *Retinal metabolism* pathway. In this regard, the transcriptomics data indicates the up-regulation of *bcmo-1* (FC = 8.5 in RNA-Seq; FC = 6.3 in RT-qPCR) and *bcmo-2* (orthologs of the human *bco1* and *bco2*), two genes directly involved in the carotenoid metabolism, catalyzing the oxidative cleavage of β-carotene into two retinal molecules, a form of vitamin A. The expression of other genes involved in the interconversion between retinal and retinol was also up-regulated (*D2063.1*, *sodh-2* and *dhrs-4*), as well as the great expression of a RBP-like retinol-binding protein (*lbp-8*, FC = 6.2), which binds extracellular retinol and transport it into the cells^57^. Vitamin A and carotenoids are especially relevant in AD progression, as several studies have demonstrated lower levels of these molecules in serum and plasma from AD patients compared with cognitively intact controls, and that the enhancement of β-carotene plasma levels is associated with better cognitive performance^59,60^. Furthermore, Aβ peptides can interfere with the retinoid acid synthesis, causing a reduction of vitamin A in tissues containing high amounts of Aβ peptides, like the brain tissue affected by AD^61^. All these results suggest that carotenoids (and probably apocarotenoids) present in DS extract are being absorbed and metabolized by *C. elegans*, which might contribute to the protection observed against paralysis.

The transcription factor enrichment analysis also suggests the activation of ELT-2 and DAF-16^42^. ELT-2 controls the gene expression changes that occur during aging^35^ and DAF-16 regulates the expression of genes involved in promoting stress resistance, the metabolism of fats, and the protection against pathogens^62^; but also, the expression of SKN-1. Apart from the stress resistance genes, DAF-16 regulated the expression of some heat shock proteins, such as *hsp-12.3* (FC = 1.8), whose up-regulation might play an important role restoring the cellular homeostasis, as other hsp proteins, such as *hsp-16.2*, can delay the paralysis and reduce Aβ oligomerization^28,63^.

Another interesting transcription factor predicted to be activated is PMK-1^39^. This transcription factor regulates the transcription factor ATF-7, which together with ELT-2 and DAF-16, regulates the expression of genes related to the host defense and innate immunity in response to intestinal infection, mediated by several C-type lectins or lysozymes^64,65^. Our results mostly agree with these observations as many C-type Lectins and genes involved in the response against pathogens are up-regulated after DS extract treatment, which could contribute to the lifespan extension in *C. elegans*. However, the relation of these genes with AD progression has not been elucidated yet. As it occurs with *skn-1*, the expression of *elt-2*, *daf-16* and *pmk-1* genes was not altered by DS extract, demonstrating the complex regulation of these transcription factors.

PQM-1 is another transcription factor suggested to be activated, and it is a key regulator of the lipid metabolism and survival^66^. Several GO terms and biological pathways related to the lipid metabolism were observed as significantly enriched after DS extract treatment, such as the β-oxidation, the sterol or the sphingolipid metabolism. *C. elegans* obtains fatty acids from its bacterial diet but it also synthesizes them de novo from acetyl CoA. In this regard, it has been demonstrated that linoleic acid (FA 18:3) increases the lifespan of treated worms and that these effects are mediated by the nhr-49 nuclear hormone receptor and the activation of the SKN-1^67^. The chemical characterization of DS extract demonstrates that FA 18:3 is the most abundant FAs, but also the presence of other unsaturated FA (FA 18:2, FA 18:1, FA 16:4) in their free or esterified forms (as TGs, DGs, acylhexosyl campesterols or TG-estolides), which could also contribute to extend the lifespan. Once absorbed, these FAs can be used to obtain energy through the β-oxidation pathway, to synthesize new FAs (such as PUFAs), or they can be incorporated into more complex molecules, such as neutral lipids or glycerophospholipids (PCs, PEs or PIs)^68^. In the case of TG molecules, FAs must be firstly liberated by triglyceride lipases, such as *lipl-1*, up-regulated after DS-extract treatment (FC = 2.9 in RNA-Seq; FC = 3.6 in RT-qPCR), and then broken down by β-oxidation. In line with this, the metabolomics and chemical enrichment analyses performed by ChemRICH after DS extract treatment of *C. elegans* indicates an increased abundance of several unsaturated TGs, PIs and PSs, which would be a consequence of the lipid enriched DS extract intake. Moreover, it is interesting to note the high accumulation of several odd-chain polyunsaturated FAs (FA 19:2, FA19:3, FA 19:4 and FA 19:5). These FAs are formed in bacteria by the addition of a methylene group across the double bond of an unsaturated FAs^69^. The two cyclopropane FAs found in *E. coli* and *C. elegans* are cis-9,10-methylenehexadecanoic acid, and cis-11,12-methyleneoctadecanoic acid, which in *C. elegans* are incorporated in TG storage lipids (or to a lesser extent in membrane phospholipids). Moreover, other complex lipids altered after DS extract treatment also contained odd-chain FAs, such TGs (53:5, 55:9 | 18:2_18:2_19:5, 57:8 | 17:1_20:3_20:4), PCs (37:6, 37:7, 39:8, 39:9, 39:10 | 19:5_20:5), PEs (33:3, 37:2, 39:6), ether-linked PEs (37:5 | 17:0_20:5, 37:1 | 19:0_18:1), LPEs (15:0, 19:1) or PIs (37:5, 37:5 | 17:0_20:5, 39:6). These results are in good agreement with our previous lipidomics study performed on neuroblastoma SH-SY5Y cells, where we observed a great increase of several unsaturated TGs, PCs and FAs (FA 18:3, 20:3, 20:4, FA 20:5) after DS extract treatment^18^. These unsaturated FAs possess neuroprotective, anti-inflammatory and anti-apoptotic properties, and the decrease of some of them have been associated with higher AD risk^70^. In addition, it has been suggested that unsaturated FAs are required for the cholinergic transmission in *C. elegans*^71^, and we have demonstrated that DS extract has a moderate anti-cholinergic capacity in vitro (20 < IC50 < 200 μg/mL)^18^. All these results suggest unsaturated FAs, in their free or esterified forms in DS extract (and the changes they exert on the lipid metabolism of *C. elegans*) as important factors contributing to the in vivo neuroprotective activity of the extract.

Apart from the lipid metabolism, the metabolomics analysis revealed two metabolites involved in the *Glutathione metabolism* pathway as altered (cadaverine and ornithine), which agrees well with the transcriptomics data; and some differences between the three groups of analyzed worms (“Control”, “DS-Treated” and “Not Induced”). For instance, the abundance of some metabolites highly accumulated in “Control” in comparison with the “Not Induced” worms were reversed in the “DS-Treated” samples. This is the case of 4-methyl-2-oxovaleric acid (or ketoleucine) and 3-methyl-2-oxovaleric acid (or ketoisoleucine) two metabolites involved in the *Valine, Leucine and Isoleucine biosynthesis* pathway. These compounds are abnormal metabolites that arises from the incomplete breakdown of branched-chain amino acids (BCAA), and a recent study has demonstrated that patients with AD (and a mouse model of AD) have elevated circulating BCAAs and their metabolites compared to healthy individuals or controls^72^. The same effects were observed for three LPEs (15:0, 18:0 and 19:1) and three PEs (37:2 | 18:1_19:1; 33:3 | 15:0_18:3 and 39:6), decreased in “DS-Treated” group in comparison to the “Control” group. In the case of LPEs, it has been suggested that high levels of LPEs result in two-fold faster median time to progression from mild cognitive impairment (MCI) to AD^73^; but also that LPE 18:1 can discriminate from early AD (preclinical + MCI) and healthy participants^74^. In other cases, the abundance of some metabolites greatly reduced in “Control” vs “Not Induced” groups were increased in “DS-Treated” group. This is the case of inosine, an intermediate in the degradation of purines and purine nucleosides, which can protect against memory impairment in AD possibly through its antioxidant and anti-inflammatory capacity^75^. The levels of other purine nucleosides (6-methyladenosine and 3’-O-methylguanosine), pyrimidine nucleosides (3’-O-methylcytidine) or related compounds (hypoxanthine) were also increased in “DS-Treated” group compared to the “Control” group, and it has been reported that there is a stage- and region-dependent deregulation of purine metabolism in AD^76^. Another group of increased metabolites are modified amino acids, such as N-methylhistidine, 3-methylhistidine, targinine (or L-NMMA), N,N-dimethylarginine (or asymmetric dimethylarginine, ADMA) and N-acetylserine; and dipeptides, some increased (Ala-Ala, Pro-hydroxyproline, Glu-Thr and gamma-Glu-Gln), others decreased (Ala-Leu and Lys-Pro). Previous studies have suggested the importance of free amino acids and dipeptides in AD pathogenesis, such as the levels of 1-mehtylhistidine (decreased in cerebrospinal fluid, plasma and urine), the levels of 3-methylhistidne (decreased in cerebrospinal fluid and plasma of AD patients) or carnosine (decreased in plasma of AD patients and demonstrated to rescue *C. elegans* AD phenotype)^77,78^. However, a more recent study has suggested that methylhistidine metabolism and carnosine synthesis are upregulated in AD patients^79^. There are also evidences suggesting the implication of altered arginine metabolism and NO pathways in the pathogenesis of AD^7,8^. In this sense, the levels of ornithine (a degradation product or arginine) have been observed as decreased in some studies^7^, or increased in other works^77^, while the levels of different methylarginines (liberated upon protein degradation), such as ADMA, are increased in plasma and decreased in cerebrospinal fluid of AD patients^80^. Moreover, it has been demonstrated that elevated ADMA contributes to the pathogenesis of AD in human cell culture models and in *C. elegans*^81^.

In summary, the present work reports the most advanced chemical characterization of a *D. salina* extract presented so far, revealing that FAs (linolenic acid, linoleic acid, palmitic acid, oleic acid and stearic acid), TGs (mainly composed of those FAs), carotenoids, apocarotenoids (10’-apo-β-carotenal, 14’-apo-β-carotenal, 13-Apo-β-carotenone and 12’-apo-β-carotenal) and glycerol, are the most abundant compounds. This extract significantly protects *C. elegans* in a dose-dependent manner against Aβ-peptide paralysis toxicity, but this effect is not related to the reduction of the intracellular ROS levels, the inhibition of Aβ gene expression or the decrease of Aβ protein aggregation. Based on the transcriptomics analysis, the neuroprotective activity of *D. salina* extract might be mediated by the alteration of several genes involved in the stress and detoxification responses, and the retinol and lipid metabolism, many of them controlled by SKN-1, ELT-2, DAF-16, PMK-1 and PQM-1 transcription factors. Complementary, the metabolomics and lipidomics analyses allowed the identification of different intracellular metabolites significantly altered, such as unsaturated FAs, LPEs, nucleosides, dipeptides and modified amino acids, which have been previously reported as beneficial during AD progression. These results combined with previous in vitro anti-inflammatory, anti-cholinesterase and neuroprotective activities (against L-glutamic acid and Aβ1-42 toxic effects) of *D. salina* extract, indicate that this extract exerts its neuroprotective capacity by different, complementary and complex mechanisms of action, making it a promising natural alternative against AD. However, future experiments using more complex in vivo models (mice) and the determination of the metabolic fate, the bioaccessibility and the bioavailability of the main bioactive compounds present in DS extract are needed to fully unravel its beneficial potential.

## Methods

### Samples and reagents

Freeze-dried *Dunaliella salina* microalgae biomass was kindly supplied by A4F - Algae for Future (Lisbon, Portugal) and stored in the dark at −20 °C in the absence of oxygen until use. For the supercritical fluid extraction, premier quality CO_2_ was provided by Carburos Metálicos (Madrid, Spain). Cholesteryl ester (CE) 22:1 was obtained from Cymit Quimica (Spain); LC-MS-grade isopropanol, ammonium formate, ammonium acetate, methyl tert-butyl ether (MTBE), toluene, Val-Tyr-Val and 2′,7′-dichlorofluorescein diacetate (DCF-DA) were obtained from Sigma-Aldrich (St Louis, MO, USA); LC-MS-grade acetonitrile (ACN), LC-MS-grade methanol, ethyl acetate and ethanol were obtained from VWR Chemicals (Barcelona, Spain), whereas Milli-Q water was obtained from a Millipore system (Billerica, MA, USA). Formic acid was purchased from Fisher Scientific (Waltham, MA, USA). The internal standard 12-[[(cyclohexylamino)-carbonyl]amino]-dodecanoic acid (CUDA) was purchased from LabClinics (Ann Arbor, MI, USA). The lipid standards lysophosphatidylcholine (LPC) 17:0, phosphatidylglycerol (PG) 17:0/17:0, ceramide (Cer) d18:1/17:0, monoacylglycerol (MG) 17:0/0:0/0:0, diacylglycerol (DG) 18:1/2:0/0:0, and triacylglycerol (TG) 17:0/17:1/17:0-d5 were provided by Avanti Polar Lipids (Alabaster, AL, USA). The isotope-labelled standards palmitic acid-d_3_, DL-alanine-3,3,3-d_3_, DL-glutamic acid-2,4,4-d_3_, d_9_-choline chloride, _15_N_2_-L-arginine. HCl and L-methionine-d_8_ were obtained from Cambridge Isotope Laboratories Inc. (Andover, MA, USA). Thioflavin-T was obtained from TCI Chemicals (Zwijndrecht, Belgium).

### Carotenoids-enriched *Dunaliella salina* extract

*Dunaliella salina* (DS) extract was prepared in a semi-pilot Speed Helix supercritical fluid extractor (Applied Separations, Allentown, PA, USA), using the same conditions as previously described to obtain the maximum extraction yield and carotenoid content^44^. Briefly, supercritical carbon dioxide was used as extracting solvent at 312.6 bar and 45.0 °C with a total extraction time set at 90 min.

### Chemical characterization of Carotenoids-enriched *Dunaliella salina* extract

DS extract was chemically characterized by HPLC coupled to a quadrupole-time-of-flight mass spectrometer (Q-TOF MS/MS) and GC coupled to a Q-TOF MS/MS spectrometer. For HPLC-Q-TOF MS/MS analysis, DS extract was diluted to 1 mg/mL in methanol containing an internal standard mixture [LPC (17:0), PG (17:0/17:0), Cer (d18:1/17:0), MG (17:0/0:0/0:0), DG (18:1/2:0/0:0), TG (17:0/17:1/17:0)-d_5_, palmitic acid-d_3_ and CUDA] and injected into a HPLC model 1290 (Agilent Technologies, Germany). Three μL were injected for ESI (+) and five μL for ESI (-), and compounds were separated using a Waters Acquity CSH C18 column (100 × 2.1 mm; 1.7 μm d_p_) equipped with a Waters Acquity VanGuard CSH C18 pre-column (5 × 2.1 mm; 1.7 μm d_p_). ACN:water (60:40 v/v, solvent A) and isopropanol:ACN (90:10 v/v, solvent B) were used as mobile phases, both containing 10 mM ammonium formate and 0.1% formic acid for ESI (+). The same mobile phases were used for ESI (-), but 10 mM ammonium acetate instead of 10 mM ammonium formate was added. The separation and MS detection were performed in the same conditions as already reported^18^. A blank sample including only the internal standards was added for blank subtraction. In the case of GC-MS, 1 mg of DS extract (and a blank sample) were dried and derivatized by adding 10 μL of methoxyamine hydrochloride in pyridine (40 mg/mL) and shaking the sample for 90 min at 30 °C. Then, the DS extract, a blank sample, and a mixture of internal standards (containing fatty acid methyl esters, FAMEs, 400505-51, Agilent Technologies, CA, USA) were trimethylsilylated by adding 90 μL of MSTFA/1% TMCS (Sigma-Aldrich,) and incubated at 37 °C for 30 min. Finally, aliquots of 1 μL of the samples were injected in splitless mode and analyzed using an Agilent 7890 GC coupled to an Agilent 7200 Q-TOF MS/MS (Agilent Technologies), equipped with an Agilent 30 m long, 0.25 mm id DB-5MS column (0.25 μm film thickness). The programmed temperature gradient started at 60 °C (1 min), and then rising at 10 °C/min to 325 °C, holding this temperature for 10 min, using a constant flow of 1 mL/min of He. Mass spectrometry data was collected using the following parameters: 750 MCP detector voltage at *m/z* 20–600 with 5 spectra/s, electron ionization at −70 eV, and ion source temperature of 250 °C.

LC-MS/MS and GC-MS raw data files were converted to ABF (Analysis Base File) format and data processing was conducted using MS-DIAL (v. 4.8) software for deconvolution, peak picking, alignment, and compound identification^82^. For LC-MS/MS data processing, the same parameters as previously reported were used^18^, and compounds were annotated following the Metabolomics Standard Initiative (MSI) guidelines^83^: MSI level 1 for compounds with precursor *m/z*, *in-house* RT-*m/z* library and MS/MS spectral library matching; MSI level 2a for compounds with precursor *m/z* and *in-house* RT-*m/z* library matching; MSI level 2b for compounds with precursor *m/z* and MS/MS spectral library matching; and MSI level 3 for compounds with precursor *m/z* matching. For GC-MS data processing the next parameters were used: retention time, 5−37.5 min; mass range, 20-600 Da; smoothing level, 2 scans; average peak width, 20 scans; minimum peak height, 1000 amplitude; mass slice width, 0.1 Da; sigma window value for deconvolution, 0.5; EI spectra cut off, 1 amplitude. Retention indices using FAMEs were used with the following parameters: retention index tolerance for MSP library identification, 3000; EI similarity cut off, 70%; identification score cut off and similarity tolerance, 70%. The MSP file used for annotation was a combination of NIST17, MassBank of North America (https://mona.fiehnlab.ucdavis.edu/spectra/browse?query=tags.text%3D%3D%22GC-MS%22) and the Fiehn BinBase DB, Rtx5-Sil MS, FAMEs RI (http://prime.psc.riken.jp/compms/msdial/main.html#MSP). Compounds were annotated as MSI level 1 for metabolites with retention time and MS spectral library matching, and MSI level 2 for metabolites with MS spectral library matching.

For HPLC-MS/MS and GC-MS data, unknown metabolites, duplicated metabolites and isotopes, metabolites with a maximum height below 1000 units and metabolites with a maximum peak height below three times the height in the blank samples were removed. Finally, the height of the different adducts from the same compound were combined.

### *C. elegans* strain and maintenance conditions

The Aβ-transgenic strain *C. elegans* CL4176 (smg-1ts[pAF29(myo-3/Ab1–42/let UTR)+pRF4(rol-6(su10069))]) was routinely propagated on nematode growth media (NGM) plates (55 mm petri dishes) with *Escherichia coli* strain OP50 as a food source. Age-synchronized populations were obtained from embryos isolated from gravid adults at 16 °C.

### Paralysis assay of Aβ-transgenic *C. elegans* CL4176 nematodes

To study the protective effect of DS extract against Aβ1–42 induced paralysis, CL4176 worms were synchronized at 16 °C in NGM (+DMSO 0.05%) or NGM supplemented with DS extract (at 1, 10, 25 or 50 μg/mL). A control condition of not induced worms (maintained at 16 °C throughout the assay) and a positive control (*Ginkgo biloba* extract EGb 761®, 100 μg/mL), were included. Paralysis was induced by up-shifting temperature from 16 °C to 25 °C so the expression of a muscle-specific Aβ1–42 was induced. Subsequently, the percentage of worms paralyzed at different times was scored, considering paralyzed if they failed to propagate a full body movement, still showing a head movement after prodding with a platinum wire. For each condition tested, two independent assays including *n* = 60 worms/assay were performed. Paralysis curves were statistically analysed using GraphPad Prism 9 software.

### Transcriptomics analyses of *C. elegans*

*C. elegans* strain CL4176 animals were cultured in NGM (+DMSO 0.05%) plates, and NGM plates supplemented with 50 μg/mL of DS extract. Aβ1–42 peptide expression was induced by an increase of temperature to 25 °C. Worms were incubated until time 26 h after paralysis induction (time at which the extract protects ca. 90% of worms from being paralyzed), and then collected and washed with M9 buffer. Four independent assays were performed for each condition. Total RNA was extracted using the Trizol® reagent method and purified using RNeasy Kit (Qiagen, Hilden, Germany). Nanodrop was used to quantify the amount and purity of RNA samples and Agilent TapeStation 4200 System was used for measuring RNA quality and integrity.

RNA samples were sequenced with the NextSeq 2000 P2 100 cycle (2×50) reagents kit on an Illumina NextSeq 2000 system (400 million reads/sample). The quality of the raw data was checked using FastQC (https://www.bioinformatics.babraham.ac.uk/projects/fastqc/) and the percentage of reads mapping to ribosomal RNA was done using riboPicker^84^. Raw reads were mapped to the *C. elegans* reference genome (Ensembl, release 104, Caenorhabditis_elegans.WBcel235.dna.toplevel.fa.gz and Caenorhabditis_elegans.WBcel235.104.gtf) (http://metazoa.ensembl.org/Caenorhabditis_elegans/Info/Index), and counted at the gene level using STAR version 2.5.3a^85^. Qualimap was used to check quality of the aligned reads^86^. Differential expression analysis was performed in the R (version 4.0.0) using the DESeq2 package version 1.28.1^87^. As the library preparation protocol was reverse stranded, the 4th column of the STAR gene counts output of each sample was taken as input for DESeq2. Genes that had less than 10 read count across all samples were filtered out before processing the data. Differentially expressed genes (DEG) were selected when the false discovery rate (FDR)-adjusted *p*-value was lower than 0.05, and the absolute log2 fold change was higher than 0.585. Category and gene ontology (GO) enrichment analysis of DEG after DS extract treatment was performed by using the WormCat 2.0 online tool (http://www.wormcat.com/)^88^, Complementary, taxon-specific gene set enrichment analysis focused on transcription factor targets was performed using WormExp^89^. Only gene sets with Bonferroni FDR < 0.05 were considered to be significant. Pathway enrichment analysis of DEG was performed by using the Joint Pathway Analysis module and the KEGG database through MetaboAnalyst 5.0 web-based software (https://www.metaboanalyst.ca/).

RT-qPCR was used to confirm the relative changes in mRNA levels of four target genes (*bcmo-1*, *gst-14*, *asm-3* and *lipl-1*) selected from RNA-Seq data sets (at 26 h after paralysis induction), and to evaluate the capability of DS extract to alter the expression of Aβ gene. Four endogenous control genes (*ama-1*, *cdc-42*, *pmp-3* and *Y45F10D.4*)^90^ were used to normalize the relative expression of the target genes in DS extract treated worms compared to control worms. Three technical replicates were performed for four biological replicates. The RNA was reverse transcribed using iScript cDNA Synthesis kit (Bio-Rad, USA) in a volume of 20 μL. RT-qPCR was then performed using the CFX Opus 384 Real Time PCR System (Bio-Rad) and SsoFast EvaGreen Supermix (Bio-Rad). The selected primers (Supplementary Table 14) were checked with Primer-BLAST of NCBI and Primer Express software of Thermo Fisher Scientific. The expression ratios and statistical significance were calculated using the Relative Expression Software Tool with a randomization test^91^.

### Intracellular levels of reactive oxidative species (ROS) in *C. elegans*

Intracellular levels of ROS were measured in *C. elegans* by using 2,7-dichlorofluorescein diacetate (DCF-DA) as previously reported^54,92^. CL4176 worms were cultured in NGM (+DMSO 0.05%) plates, and NGM plates supplemented with 50 μg/mL of DS extract. After 26 h of paralysis induction, worms were collected, washed with M9 buffer, and transferred into a microfuge tube. Worms were pelleted by centrifugation at 14,000 rpm for 2 min, sonicated with a Branson Digital Sonifier 450 (Branson Ultrasonics Corporation, CT, USA) at 30% amplitude (4 cycles of 15 s on/15 s off), and total protein concentration was quantified using the Bradford method. Equal amount of protein from five biological replicates (and three technical replicates) were mixed with PBS in a final volume of 200 μL with 50 μM DCF-DA. Fluorescence was acquired in a BioTek Cytation 5 plate reader (Agilent, USA) using excitation at 485 nm and emission at 530 nm.

### Staining of Aβ aggregates with thioflavin-T in *C. elegans* CL4176

Aβ protein aggregation was measured in *C. elegans* by using thioflavin-T stain as previously described^54^. Equal amount of protein from the experiment of the previous section (five biological replicates and three technical replicates) were mixed with 2 μl of 1 mM thioflavin-T in a final volume of 100 μl of PBS. Fluorescence was measured using the BioTek Cytation 5 plate reader with excitation at 440 nm and emission at 482 nm.

### Metabolomics analyses of *C. elegans*

Worms of the transgenic *C. elegans* strain CL4176 were cultured in NGM (+DMSO 0.05%) plates, and NGM plates supplemented with 50 μg/mL of DS extract. Aβ1–42 peptide expression was induced by an increase of temperature to 25 °C. A control condition of not induced worms (maintained at 16 °C throughout the assay) was included. Worms were incubated until time 26 h after paralysis induction (time at which the extract protects ca. 90% of worms from being paralyzed), and then collected and washed with M9 buffer. Five independent assays were performed for each condition.

Extraction of intracellular metabolites was carried out using a biphasic solvent system consisting on cold methanol, MTBE, and water. The procedure was similar as in Gallego et al. (2022)^18^, but 350 μL of the upper layer was collected for the analysis of non-polar compounds using CSH-Q-TOF MS/MS, and 125 μL of the bottom layer was collected twice for the analysis of polar compounds by using HILIC-Q-TOF MS/MS and GC-Q-TOF MS. Finally, every fraction was evaporated to dryness. The dried non-polar fractions were resuspended in 110 μL of methanol:toluene (9:1, v/v) mixture containing 50 ng/mL of CUDA and analyzed using the same conditions as for the chemical characterization of the DS extract (see above). Method blanks and pooled mixtures of all samples were included as quality control samples. The first dried polar fractions were resuspended in 80 μL of ACN:water (4:1, v/v) with a mixture of internal standard compounds (CUDA, DL-alanine-d_3_, DL-glutamic acid-d_3_, d_9_-choline chloride, _15_N_2_-L-arginine, L-methionine-d_8_) and Val-Tyr-Val for their analysis by HILIC-Q-TOF MS/MS following a previously optimized approach^18^. The second dried polar fractions were derivatized using the same protocol as for the chemical characterization of the DS extract (see above), but a mixture of FAMEs was added to every sample. Samples analysis were performed using the same GC-Q-TOF MS instrument and conditions as specified above. Method blanks and pooled mixtures of all samples were included as quality control samples.

Quality control for each of the above analyses was assured by: (i) randomization of the sequences; (ii) injection of extraction blanks and pool mixture samples to equilibrate the LC-MS or GC-MS systems before, during and after the different sequences; (iii) checking retention times, peak shapes and the intensities of the spiked internal standards; and (iv) monitoring the mass accuracy of internal standards during the run.

All raw data files were converted to ABF format and data of each analytical platform were processed separately by MS-DIAL (v. 4.8). For CSH-Q-TOF MS/MS and GC-Q-TOF MS data, the parameters used were the same as for the characterization of the DS extract (see above). In the case of HILIC-Q-TOF MS/MS data processing, the parameters previously optimized were used^18^. The MSP file used for annotation was generated by combining MS/MS spectra from NIST20 MS/MS database, the LipidBLAST mass spectral library^93^, and MassBank of North America (https://mona.fiehnlab.ucdavis.edu/downloads).

### Data post-processing and statistical analysis

The lists of metabolites obtained from each analytical platform (CSH-Q-TOF MS/MS, HILIC-Q-TOF MS/MS and GC-Q-TOF MS) and ionization mode (ESI (+) and ESI (-)) were post-processed independently. Missing values were imputed by half of the minimum value, and data were processed using MS-FLO tool (https://msflo.fiehnlab.ucdavis.edu/). Duplicated metabolites and isotopes were detected and manually filtered, and the height of the different adducts from the same compound were combined. The set of metabolites were normalized by using the sum peak height of all identified metabolites in the analyses (mTIC). The datasets from each platform and ionization modes were then combined to generate a joined dataset. Data with the lowest RSD in the pooled samples were retained.

Principal Component Analysis (PCA) was performed by using MetaboAnalyst 5.0 web-based software (https://www.metaboanalyst.ca/), with previous “Auto-scale” normalization. ANOVA was used to identify significantly altered metabolites between the three groups of samples with FDR lower than 0.05. Two-sample *T*-Test was applied to identify significantly altered metabolites between the different conditions studied, and applying an FDR lower than 0.05 and a fold change (FC) cut-off filter (0.67 > FC > 1.5). MetaboAnalyst 5.0 was used to identify overrepresented biological pathways in the lists of significantly altered metabolites, and chemical similarity enrichment analysis was performed using the ChemRICH bioinformatic tool (https://chemrich.fiehnlab.ucdavis.edu/).

### Reporting summary

Further information on research design is available in the Nature Research Reporting Summary linked to this article.

### Supplementary information

Supplementary Figures

Reporting summary

Supplementary Tables

## Data Availability

The authors declare that the RNA-Seq data that support the findings of this study are available for public in the Sequence Read Archive (SRA) with BioProject ID PRJNA960634. The metabolomics data are available within the manuscript and supplementary information files, and raw data can be provided by the corresponding author on reasonable request.
