# Supplementary material for: In vivo neuroprotective capacity of a Dunaliella salina extract - comprehensive transcriptomics and metabolomics study
Source: NPJ Sci Food. 2024 Jan 10;8:4. doi: 10.1038/s41538-023-00246-7 (PMC10782027; doi:10.1038/s41538-023-00246-7)
Supplement: Supplementary file 1 — Supplementary Figures [file 41538_2023_246_MOESM1_ESM.pdf]

Supplementary Figures for

In vivo neuroprotective capacity of a *Dunaliella salina* extract - Comprehensive transcriptomics and metabolomics study

Alberto Valdés[1]*, José David Sánchez-Martínez[1], Rocío Gallego[1], Elena Ibáñez[1], Miguel Herrero[1] and Alejandro Cifuentes[1]

[1] Laboratory of Foodomics, Institute of Food Science Research (CIAL, CSIC-UAM), Calle Nicolás Cabrera 9, 28049 – Madrid, Spain.

* Corresponding author: a.valdes@csic.es; Tel.: +34-910017900 Ext. 439371

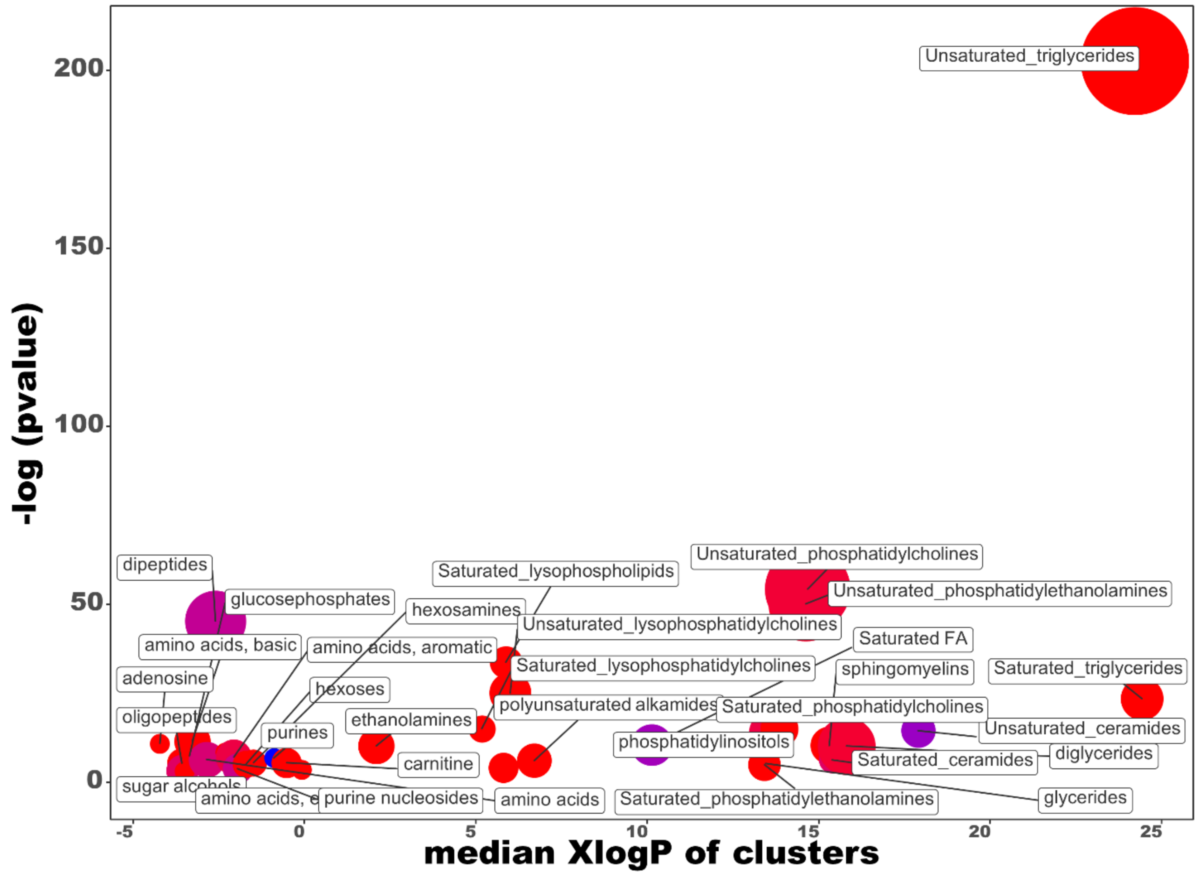

Supplementary Figure 1. Chemical similarity enrichment results of metabolomics data from *Caenorhabditis elegans* in "Control" conditions (0.05% DMSO) compared to "Not Induced" conditions (0.05% DMSO and maintained at 16 °C throughout the assay) for 26 h, and obtained using ChemRICH. Statistical enrichment analysis utilized chemical similarity and ontology mapping to generate metabolite clusters. The y-axis shows the most significantly altered clusters on top; the x-axis shows the XlogP values of clusters. Cluster colors give the proportion of increased or decreased compounds (red = increased, blue = decreased) in each cluster. Chemical enrichment statistics is calculated by Kolmogorov-Smirnov test. Only enrichment clusters are shown that are significantly different at $p < 0.05$**.**
