# Supplementary material for: In vivo neuroprotective capacity of a Dunaliella salina extract - comprehensive transcriptomics and metabolomics study
Source: NPJ Sci Food. 2024 Jan 10;8:4. doi: 10.1038/s41538-023-00246-7 (PMC10782027; doi:10.1038/s41538-023-00246-7)
Supplement: Supplementary file 2 — Reporting summary [file 41538_2023_246_MOESM2_ESM.pdf]

nature portfolio

Corresponding author(s): Alberto Valdés

Last updated by author(s): Nov 19, 2023

Reporting Summary

Nature Portfolio wishes to improve the reproducibility of the work that we publish. This form provides structure for consistency and transparency in reporting. For further information on Nature Portfolio policies, see our Editorial Policies and the Editorial Policy Checklist.

Statistics

For all statistical analyses, confirm that the following items are present in the figure legend, table legend, main text, or Methods section.

n/a	Confirmed	
☐	☒	The exact sample size (n) for each experimental group/condition, given as a discrete number and unit of measurement
☐	☒	A statement on whether measurements were taken from distinct samples or whether the same sample was measured repeatedly
☐	☒	The statistical test(s) used AND whether they are one- or two-sided<br>*Only common tests should be described solely by name; describe more complex techniques in the Methods section.*
☐	☒	A description of all covariates tested
☐	☒	A description of any assumptions or corrections, such as tests of normality and adjustment for multiple comparisons
☐	☒	A full description of the statistical parameters including central tendency (e.g. means) or other basic estimates (e.g. regression coefficient) AND variation (e.g. standard deviation) or associated estimates of uncertainty (e.g. confidence intervals)
☐	☒	For null hypothesis testing, the test statistic (e.g. F, t, r) with confidence intervals, effect sizes, degrees of freedom and P value noted<br>*Give P values as exact values whenever suitable.*
☒	☐	For Bayesian analysis, information on the choice of priors and Markov chain Monte Carlo settings
☒	☐	For hierarchical and complex designs, identification of the appropriate level for tests and full reporting of outcomes
☒	☐	Estimates of effect sizes (e.g. Cohen's d, Pearson's r), indicating how they were calculated

Our web collection on statistics for biologists contains articles on many of the points above.

Software and code

Policy information about availability of computer code

Data collection	For RNA-Seq data: NextSeq 1000/2000 Control Software Suite v1.4.0.39521.<br>For RT-qPCR: CFX Opus 384 Real Time PCR System (Bio-Rad).<br>For intracellular ROS and Ab protein aggregates: BioTek Gen5 (v3.10)<br>For metabolomics data: Agilent MassHunter Workstation LC-MS Data Acquisition (v10.1), Agilent MassHunter Workstation GC-MS Data Acquisition (v10.1.49)
Data analysis	For RNA-Seq data: FastQC (v0.12.1), riboPicker (https://ribopicker.sourceforge.net/), STAR (v2.5.3a), Qualimap 2, R (v4.0.0), DESeq2 (v1.28.1), WormCat 2.0 (http://www.wormcat.com/), WormExp (https://wormexp.zoologie.uni-kiel.de/wormexp/), MetaboAnalyst 5.0 (https://www.metaboanalyst.ca/).<br>For RT-qPCR: REST 2009.<br>For intracellular ROS and Ab protein aggregates: Microsoft Excel 2019.<br>For metabolomics data: Agilent MassHunter Workstation Qualitative Analysis (v10.0), AbfConverter(v4.0.0), MS-DIAL (v4.8), MS-FLO tool (https://msflo.fiehnlab.ucdavis.edu/), MetaboAnalyst 5.0 (https://www.metaboanalyst.ca/), ChemRICH (https://chemrich.fiehnlab.ucdavis.edu/).

For manuscripts utilizing custom algorithms or software that are central to the research but not yet described in published literature, software must be made available to editors and reviewers. We strongly encourage code deposition in a community repository (e.g. GitHub). See the Nature Portfolio guidelines for submitting code & software for further information.

Data

Policy information about availability of data

All manuscripts must include a data availability statement. This statement should provide the following information, where applicable:

- Accession codes, unique identifiers, or web links for publicly available datasets
- A description of any restrictions on data availability
- For clinical datasets or third party data, please ensure that the statement adheres to our policy

The authors declare that the RNA-Seq data that support the findings of this study are available for public in the Sequence Read Archive (SRA) with BioProject ID PRJNA960634. The metabolomics data are available within the manuscript and supplementary information files, and raw data can be provided by the corresponding author on reasonable request.

Research involving human participants, their data, or biological material

Policy information about studies with human participants or human data. See also policy information about sex, gender (identity/presentation), and sexual orientation and race, ethnicity and racism.

Reporting on sex and gender	N/A
Reporting on race, ethnicity, or other socially relevant groupings	N/A
Population characteristics	N/A
Recruitment	N/A
Ethics oversight	N/A

Note that full information on the approval of the study protocol must also be provided in the manuscript.

Field-specific reporting

Please select the one below that is the best fit for your research. If you are not sure, read the appropriate sections before making your selection.

☒ Life sciences ☐ Behavioural & social sciences ☐ Ecological, evolutionary & environmental sciences

For a reference copy of the document with all sections, see nature.com/documents/nr-reporting-summary-flat.pdf

Life sciences study design

All studies must disclose on these points even when the disclosure is negative.

Sample size	For each condition tested in the paralysis assay, two independent experiments including n = 60 worms/assay were performed. For transcriptomics and metabolomics experiments, four independent experiments for each group were performed.
Data exclusions	No data were excluded.
Replication	All attempts at replication were successful.
Randomization	All samples were allocated randomly.
Blinding	Not applicable for this study because our aim was to measure the paralysis effects of Dunaliella salina microalgae extract compared to control conditions, and to investigate the transcriptomics and metabolomics changes produced after the treatment, so we need to know which sample belongs to which group to perform the analyses.

Reporting for specific materials, systems and methods

We require information from authors about some types of materials, experimental systems and methods used in many studies. Here, indicate whether each material, system or method listed is relevant to your study. If you are not sure if a list item applies to your research, read the appropriate section before selecting a response.

Materials & experimental systems

n/a	Involved in the study
☒	☐ Antibodies
☒	☐ Eukaryotic cell lines
☒	☐ Palaeontology and archaeology
☐	☒ Animals and other organisms
☒	☐ Clinical data
☒	☐ Dual use research of concern
☒	☐ Plants

Methods

n/a	Involved in the study
☒	☐ ChIP-seq
☒	☐ Flow cytometry
☒	☐ MRI-based neuroimaging

Animals and other research organisms

Policy information about studies involving animals; ARRIVE guidelines recommended for reporting animal research, and Sex and Gender in Research

Laboratory animals	Aβ-transgenic strain Caenorhabditis elegans CL4176 (smg-1ts[pAF29(myo-3/Ab1–42/let UTR)+pRF4(rol-6(su10069))])
Wild animals	The study did not involve wild animals.
Reporting on sex	The experiments were performed with hermaphrodite animals.
Field-collected samples	The study did not involve samples collected from the field.
Ethics oversight	No ethical approval or guideance is required for working with Caenorhabditis elegans.

Note that full information on the approval of the study protocol must also be provided in the manuscript.
